# Differentiated embryonic chondrocyte-expressed gene 1 is associated with hypoxia-inducible factor 1α and Ki67 in human gastric cancer

**DOI:** 10.1186/1746-1596-8-37

**Published:** 2013-02-27

**Authors:** Yan-Fei Jia, Dong-Jie Xiao, Xiao-Li Ma, Yan-Yan Song, Rui Hu, Yi Kong, Yan Zheng, Shu-Yi Han, Ruan-Li Hong, Yun-Shan Wang

**Affiliations:** 1 Central Laboratory, Jinan Central Hospital Affiliated to Shandong University, Jinan, 250013,, Shandong Province, China; 2 Shandong Province Key Lab of Tumor Target Molecule, Jinan Central Hospital Affiliated to Shandong University, Jinan, 250013,, Shandong Province, China; 3 Shandong University School of Medicine, Jinan, 250012,, Shandong Province, China; 4 Gynecology and obstetrics, Jinan Central Hospital Affiliated to Shandong University, Jinan, 250013,, Shandong Province, China

**Keywords:** Differentiated embryo chondrocyte 1, Hypoxia-inducible factor 1α, Proliferation, Gastric cancer, Immunohistochemistry

## Abstract

**Background:**

Gastric cancer is a leading causes of cancer-related deaths ,but the underlying molecular mechanisms of its progression are largely unknown. Differentiated embryonic chondrocyte-expressed gene 1 (DEC1), is an important transcription factor involved in the progression of tumors and has recently been identified to be strongly inducible by hypoxia. Little is known about the contribution of DEC1 to the intracellular hypoxia and proliferation signaling events in gastric cancer.

**Methods:**

Immunohistochemistry was used to detect the expression of DEC1, hypoxia-inducible factor 1(HIF-1α) and Ki67 in 173 human gastric cancer samples and adjacent non-tumor tissues samples. The relationship between DEC1, HIF-1α and Ki67 was evaluated.

**Results:**

DEC1 protein was persistently expressed in the nucleus and cytoplasm of gastric cancer tissue. The protein expression of DEC1 and HIF-1α in tumour tissues was 83.8% and 54.3%, respectively, and was significantly higher than that in adjacent normal tissues (83.8% vs 23.7%, P <0.001; 54.3% vs 12.7%, P< 0.001). The expression of DEC1 and HIF-1α was associated with poor histological differentiation. (*P* < 0. 01). Furthermore, DEC1 level was positively correlated with HIF-1α (P < 0. 01, r=0.290) and Ki67 expression (P < 0. 01, r=0.249).

**Conclusion:**

The upregulation of DEC1 may play an important role in hypoxia regulation and cell proliferation in gastric cancer. The relevant molecular mechanism requires further investigation.

**Virtual Slides:**

The virtual slide(s) for this article can be found here: http://www.diagnosticpathology.diagnomx.eu/vs/1794565980889391med.motic.com/MoticGallery/Slide?id=08d180cd-5fdb-4cee-830a-0b1fef3311f2&user=2C69F0D6-A478-4A2B-ABF0-BB36763E8025med.motic.com/MoticGallery/Slide?id=4762991d-3f2f-43aa-b4bf-ecdd2c2ae3ec&user=2C69F0D6-A478-4A2B-ABF0-BB36763E8025med.motic.com/MoticGallery/Slide?id=2717f209-b3fd-4e71-b621-0d60ea507a82&user=2C69F0D6-A478-4A2B-ABF0-BB36763E8025

## Background

Human differentiated embryonic chondrocyte-expressed gene 1 (DEC1) (also known as Stra13/Bhlhb2/Sharp2) is a basic helix-loop-helix (bHLH) transcription factor. Although little is known about its function in human physiological and pathological processes, recent studies have shown that DEC1 is a hypoxia-induced gene involved in cell differentiation, proliferation, and apoptosis [1,2].

Miyazaki K et al. [3] identified the hypoxia response elements in the DEC1 promoter and found that the DEC1 gene is a direct target of hypoxia-inducible factor 1 (HIF-1). DEC1 may be a hypoxia-regulated gene, so its expression in human tumors may be a direct marker of tumor hypoxia. The association of the clinical value of DEC1 expression and HIF-1α, the endogenous marker of hypoxia, has not been reported for gastric cancer. The studies with cultured cell lines have revealed that DEC1 expression is associated with hypoxia and HIF-1α protein expression, and blockage of the HIF-1α protein led to reduced DEC1 expression [4].

The clinical progression of gastric cancer is rapid and is reflected by various proliferation markers. The role of DEC1 in cell proliferation is inconsistent. DEC1 inhibits apoptosis in human embryonic kidney 293 cells [5,6] and induces apoptosis in mouse fibroblast NIH3T3 cells [7]. In addition, DEC1 has pro-apoptotic properties in human breast cancer cells [8].The intracellular signaling events related to the role of DEC1 in gastric cancer have not been well established.

In this study, we used immunohistochemistry to evaluate the expression status and clinicopathological features of DEC1 and HIF-1α in 173 human gastric cancer samples and its adjacent non-tumor tissues by immunohistochemistry. The associations between DEC1 and HIF-1α, and proliferation marker Ki67 were analyzed.

## Materials and methods

### Patients and samples

Surgically resected gastric cancer specimens were collected from 173 patients (117 males) at Jinan Central Hospital Affiliated to Shandong University, China, January 2011 to June 2012. The mean age of the patients was 62 years (range 30–88 years). Among the 173 tumor samples, 53 were well or moderately differentiated and 120 were poorly differentiated. The formalin-fixed, paraffin-embedded samples were retrospectively and randomly selected from the files of the Department of Pathology after the protocol was approved by the local research ethics committee. All the samples were evaluated for diagnosis by 2 experienced pathologists for diagnosis. None of the patients had received chemotherapy or radiation therapy preoperatively.

### Immunohistochemistry

The tissues were fixed in 10% neutral buffered formalin for 12 h and were routinely processed. The paraffin wax-embedded tissue blocks were cut into 4-μm-thick sections. The formalin-fixed, paraffin-embedded sections were heated at 60°C for 60 min and placed into xylene for deparaffinization and graded ethanol for rehydration, then washed in phosphate-buffered saline (PBS). The antigen retrieval involved the use of a prewarmed pressure cooker with a solution of antigen retrieval citrate buffer (pH 6.8) for 3 min. Following de-pressurization, cold water was poured into the cooker for 10 min, then sections were rinsed well in warm water. The sections were incubated in 3% H_2_O_2_ in methanol for 10 min, then washed 3 times with PBS and incubated with the primary antibodies rabbit polyclonal anti-DEC1 (1:50, Genetex, CA, USA), mouse monoclonal anti-HIF-1α (1:50, Santa Cruz Biotechnology, CA, USA), or rabbit monoclonal anti-Ki67 (1:50, Cell Signaling Technology, MA, USA) overnight in a moist chamber at 4°C. Following a final wash, the sections were incubated with secondary antibody (KIT-5010, Max Vision, Maixin.Bio, China); TBS-Tween20 was used in all dilutions and intervening rinses. The ections were allowed to develop in diaminobenzidine (DAB) for 5 min, and then were counterstained with hematoxylin. The slides were viewed under a microscope. The sections incubated without primary antibody were negative controls included in each run.

### Immunohistochemical staining evaluation

The assessment of DEC1, HIF-1α was as previously described [9,10], by percentage of stained tumor cells and staining intensity. We examined a minimum of 3 different high-power (× 400) fields of tumor infiltration. The percentage of positive tumor cells was rated as follows: 0, staining in ≤10% of tumor cells; 1+, weak staining in >10% of cells; 2+, moderate staining in >10% of cells; 3+, strong moderate staining in >10% of cells. Tumors scored as 3+/2+ were positive cases; tumors scored as 0/1+ were designated as negative cases. The Ki67 scoring system was 0 assigned for no cells staining positively; 1 for ≤ 10% of cells staining positively; 2 for > 10% of cells staining positively. The tumors scored as 3+/2+ were positive cases; the tumors scored as 0/1+ were designated as negative cases. Expression was analyzed by 2 independent investigators who used a multiheaded microscope and were blinded to the clinical data with consensus.

### Statistical analysis

The statistical analysis used SPSS v11.5 (SPSS Inc., Chicago, IL). A chi-square test was used to test the association of DEC1 and HIF-1α expression and the clinicopathological variables. The spearman correlation analysis was used to assess the correlation between DEC1 expression and HIF-1α and Ki67 expression. *P* < 0.05 was considered statistically significant.

## Results

### Expression of DEC1 and HIF-1α in gastric cancer and adjacent non-tumour tissues

**Figure 1:**
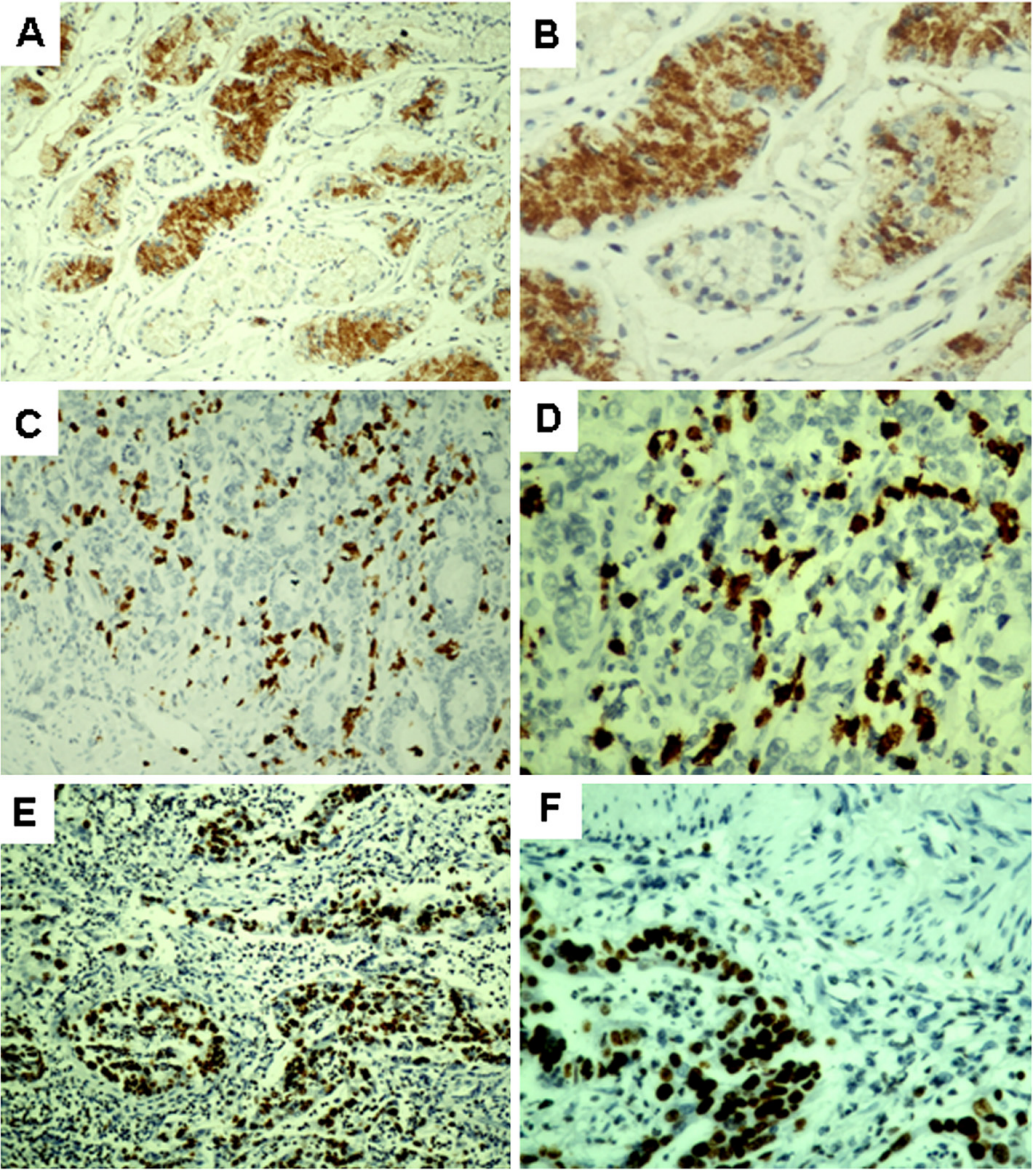
**Expression of differentiated embryonic chondrocyte-expressed gene 1 (DEC1), hypoxia-inducible factor 1α (HIF-1α) and Ki67 protein in human gastric cancer tissue.** Brown shows positive expression. Poorly differentiated gastric cancer with nuclear and cytoplasmic staining for DEC1 (**A:** SP, × 100; **B:** SP, × 400). Poorly differentiated gastric cancer with nuclear staining for HIF-1α (**C:** SP, × 100; **D:** SP, × 400). Poorly differentiated gastric cancer with nuclear staining for Ki67 (**E:** SP, × 100; **F:** SP, × 400).

DEC1 protein expression was located in the nucleus and cytoplasm of gastric cancer cells and was diffuse in 145 of 173 specimens (83.8%; Table 1, Figure 1A and B). Compared with the gastric cancer tissue, the adjacent non-tumour tissues showed reduced DEC1 expression (23.7%, P <0.001), with only cytoplasmic positive staining. The proportion of positive DEC1 nuclear and/or cytoplasmic staining in well and moderately differentiated tissues was 67.9% (36/53) and in poorly differentiated tissues 90.8% (109/120), with a significant difference found between the two. The total DEC1 protein staining was associated with poorly differentiated histological grade (P <0.01; Table 2). DEC1 expression did not differ by tumor size, lymph node metastasis, tumor-node-metastasis stage, or age or sex of the patients.

**Table 1:** **Expression of differentiated embryo chondrocyte 1 and hypoxia-inducible factor 1**α **protein in gastric cancer and adjacent non-tumor tissues n (%)**

	**Cases**	**DEC1 protein**		**HIF-1α protein**	
		**Positive**	**P value**	**Positive**	**P value**
Gastric cancer	173	145(83.8%)	0.000	94(54.3%)	0.000
Adjacent non-tumor tissues	173	41(23.7%)		22(12.7%)	

*DEC1* Differentiated embryo chondrocyte 1, *HIF-1a* Hypoxia-inducible factor-1.

**Table 2:** Correlation between the expression of DEC1 and clinicopathological factors

**Clinicopathologic features**	**DEC1 protein expression**		**P value**	**HIF-1α protein expression**		**P value**
	**Negative**	**Positive**		**Negative**	**Positive**	
Age (yr)			0.108			0.463
≤50	8	23		16	15	
>50	20	122		63	79	
Sex			0.082			0.136
Male	15	102		58	59	
Female	13	43		21	35	
Tumor size (cm)			0.283			0.920
≤5 cm	8	57		30	35	
>5 cm	20	88		49	59	
Lymph node metastasis			0.082			0.000
Yes	24	101		44	81	
No	4	44		35	13	
TNM stage			0.397			0.013
I~II	9	59		39	29	
III~IV	19	86		40	65	
Tumor differentiation status			0.000			0.004
Well and moderate	17	36		33	20	
Poor	11	109		46	74	

Nuclear expression of HIF-1α was found in 54.3% of the tumors, which was significantly higher than that in the adjacent normal tissues (54.3% vs 12.7%, P < 0.01, Table 1, Figure 1C and D). Furthermore, we also found that the expression of HIF-1α was correlated with lymph node metastasis, TNM stage and poorly differentiated histological grade (P < 0.05, Table 2).

### Correlation of DEC1 and HIF-1α expression in human gastric cancer

To study whether DEC1 is associated with HIF-1α in gastric cancer, we performed a correlative analysis. The results showed that there is a significant correlation between nuclear or cytoplasmic DEC1 expression and nuclear HIF-1α expression (P <0.01, r=0.290; Table 3).

**Table 3:** **Correlation between the expression of DEC1 and HIF-1**α

	**DEC1 expression**		***P *value**	***r *value**
	**Positive**	**Negative**		
	**(n = 145)**	**(n = 28)**		
HIF-1α expression			<0.001	0.290
Positive (*n* = 94)	88	6		
Negative (*n* = 79)	57	22		

### Correlation of DEC1 and Ki67 expression in human gastric cancer

In all, 56.9% (98/173) of gastric cancer specimens expressed Ki67 (Figure 1E and F). DEC1 and Ki67 levels were significantly correlated (P <0.01, r = 0.249; Table 4).

**Table 4:** Correlation between the expression of DEC1 and Ki67

	**DEC1 expression**		**P value**	***r *value**
	**Positive**	**Negative**		
	**(n = 145)**	**(n = 28)**		
Ki67 expression			0.001	0.249
Positive (*n* = 98)	90	8		
Negative (*n* = 75)	55	20		

## Discussion and conclusions

Previous reports of the implications of DEC1 overexpression in cancer have been inconsistent [10-12], so we investigated gastric cancer specimens from a large consecutive series of gastric cancer patients who had undergone similar surgeries. We extended previous studies by finding a positive correlation between DEC1 and HIF-1α expression and cancer proliferation.

We found that the DEC1 protein was persistently expressed in both the cytoplasm and the nucleus of gastric cancer. DEC1 was also widely expressed in the cytoplasm of other human tumours, including breast cancers, colon/pancreas adenoma, and ductal carcinomas [1]. Only nuclear DEC1 reactivity was found in some clinicopathological studies [1,13]. These differences might be explained by the status of the paraffinized sections, subjective interpretation by pathologists, the production of antibodies or the variation in techniques. Although a transcription factor, nuclear DEC1 is likely to be an active form, which is synthesized and degraded in the cytoplasm. Its expression may be redistributed during tissue collection, which would be difficult to control, but the overall expression indicates upregulation of the pathway in gastric cancer. In this study, positive DEC1 staining was found in 145 of 173 (83.8%) specimens, and nuclear/cytoplasmic DEC1 expression was found to be significantly correlated with nuclear HIF-1α expression. The analysis based on pure nuclear expression showed very marginal or no statistical association with HIF-1α, showing that strong cytoplasmic DEC1 expression, which is a tumourspecific finding, better reflects the DEC1 regulated pathway in paraffin-embedded material.

HIF-1α is a well-established molecule induced under hypoxic conditions [14]. As well, DEC1 is inducible by HIF-1α in various cancer cell lines [5,15-25]. The mechanism of this interaction has been shown to be the HIF-1α/β complex directly binding to the HIF-1 binding site in the hypoxia response element of the DEC1 promoter, which initiates the transcription of DEC1 [3,17,26]. Clinical studies revealed a possible association of HIF-1α and DEC1 expression by immunohistochemical staining in solid cancers such as non-small lung cancer and breast cancer [1,2]. HIF-1-dependent hypoxia-induced DEC1 expression may be in a hypoxic status in solid cancers. Some studies have shown that the promotion or inhibition of cell differentiation by DEC1 is cell-type specific and differs in different original tissues. Overexpression of Stra13 inhibits mesodermal differentiation but promotes neuronal differentiation in P19 cells [27]. DEC1 was found to be associated with the hypoxic response and high tumour grade in human breast cancers [13]. In hepatocellular carcinoma, the expression of nuclear DEC1 was greater in well differentiated than in poorly differentiated malignant hepatocellular carcinoma [15]. Furthermore, a positive association between tumour grade and HIF-1α has been reported [28]. These observations suggest that tumor hypoxia can inhibit cancer cell differentiation, and DEC1, induced by HIF-1α, may function as a HIF-1α effector to mediate the effect of hypoxia on cell differentiation. Thus, DEC1 and HIF-1α expression is correlated with poor differentiation, as we describe, and is in accordance with the induction by HIF-1α and the ability of DEC1 to repress tumor differentiation.

**Figure 2:**
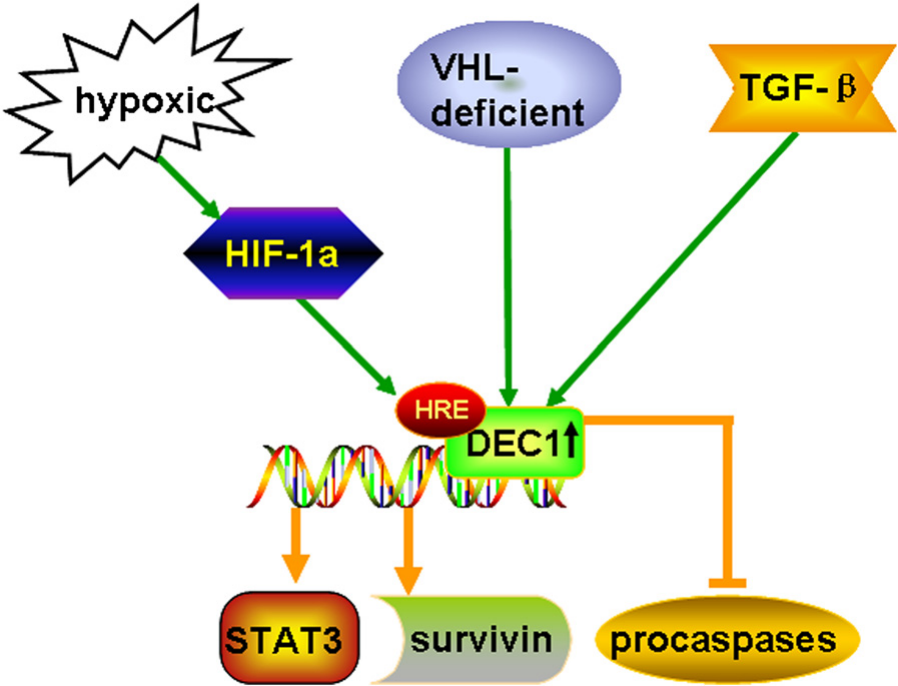
**Schematic representation of promotion function of DEC1 in cell proliferation.** DEC1 is inducible by HIF-1α in cancer cell. The mechanism of this interaction has been shown to be the HIF-1α/β complex directly binding to the HIF-1 binding site in the hypoxia response element(HRE) of the DEC1 promoter to initiate the transcription of DEC1.The cells that lack the functional tumor suppressor von Hippel-Lindau(VHL) express higher levels of DEC1. DEC1 is a downstream target of transforming growth factor β signaling, which promotes the survival of cells. DEC1 transcriptionally upregulates the expression of the survivin and signal transducer and activator of transcription 3 (STAT3). Forced expression of DEC1 selectively inhibits the activation of procaspases.

Dysregulation of cellular proliferation is a prominent feature of cancers. Ki67 nuclear antigen is a proliferative marker and is a good indicator of the proliferative and differentiation ability of gastric carcinoma cells [29]. DEC1 upregulation encourages the growth of malignant cells by increasing cell proliferation (Figure 2). Cells that lack the functional tumor suppressor von Hippel-Lindau express higher levels of DEC1 [26]. DEC1 is a downstream target of transforming growth factor β signaling [17], which promotes the survival of breast cancer cells. Forced expression of DEC1 antagonizes serum deprivation-induced apoptosis and selectively inhibits the activation of procaspases [5]. DEC1 transcriptionally upregulates the expression of the survivin [6] and signal transducer and activator of transcription 3 [30], providing a molecular explanation for DEC1 anti-apoptosis. In contrast, DEC1 induces apoptosis in mouse fibroblast NIH3T3 cells, possibly through the regulation of p53 [7]. DEC1 may play a key role in signaling pathways that lead to growth arrest and terminal differentiation by repressing target genes via histone deacetylase-dependent and -independent mechanisms [31]. DEC1 overexpression induces the apoptosis of breast cancer MCF-7 cells [8]. DEC1 also acts as a transcriptional repressor to inhibit the transcription of cyclin D1, which is associated with cell proliferation and tumorigenesis [32]. The promotion or inhibition function of DEC1 in cell proliferation may depend on the cell type. In our study, DEC1 positively correlated with the cell proliferation marker Ki67. The increased expression of HIF-1α DEC1, and Ki67 in poorly differentiated tissues implied that the tumor cells lost growth control in gastric carcinogenesis, thus leading to a DNA synthesis disorder, which reflected the malignant behavior of tumor cells.

In this study, we did not find any association between lymph node metastasis, TNM stage and DEC1 expression, which was in agreement with a study by Chakrabarti J et al. [13] study. Nevertheless, upregulation of DEC1 is strongly associated with HIF-1a and Ki67,previously shown to be associated with aggressive clinical behavior [9,28,33,34], which supports that DEC1 is a marker of activated hypoxic pathways and a novel biomarker of clinical aggressiveness. Although further investigations are needed to clarify the molecular mechanisms involved in DEC1 overexpression, DEC1 may be a valuable therapeutic target in gastric cancer.

## Competing interests

The authors declare that they have no competing interests.

## Authors’ contributions

YJ, YS, HR, RH did the immunohistochemical analysis. YF and XM reviewed all the pathological slides. YJ, DX, YZ, HY, YK analyzed the data. YW designed the study. YJ drafted the manuscript. All authors read and approved the final manuscript.
